# Colorimetric and fluorescent probes for real-time naked eye sensing of copper ion in solution and on paper substrate

**DOI:** 10.1098/rsos.171161

**Published:** 2017-11-08

**Authors:** Dugang Chen, Pengyu Chen, Luyi Zong, Yimin Sun, Guangchao Liu, Xianglin Yu, Jingui Qin

**Affiliations:** 1 Key Laboratory for Green Chemical Process of Ministry of Education, School of Chemical Engineering and Pharmacy, Wuhan Institute of Technology, Wuhan 430205, People's Republic of China; 2 School of Materials Science and Engineering, Wuhan Institute of Technology, Wuhan 430205, People's Republic of China; 3 College of Chemistry and Molecular Science, Wuhan University, Wuhan 430072, People's Republic of China

**Keywords:** colorimetric probe, fluorescent probe, copper ion, solid-state sensor

## Abstract

In this paper, BT ((*E*)-2-(4-(4-(bis(pyridin-2-ylmethyl)amino)styryl)-3-cyano-5,5-dimethylfuran-2(5H)-ylidene)malononitrile) with strong donor–π-acceptor structure was synthesized, which showed both colorimetric and fluorescent sensing ability toward Cu^2+^ with high selectivity and sensitivity. Job plot and mass spectra measurement revealed a 1 : 1 coordination mode between Cu^2+^ and probe BT in ethanol/HEPES (1 : 4 v/v) buffer (pH 7.2) solution, and the binding constant was calculated to be 3.6 × 10^4^ M^–1^. The colour of BT solution (10 µM) immediately turned from purple red to yellow and the red fluorescence was quenched obviously when a certain amount of Cu^2+^ was added, which enabled a dual-channel detection of Cu^2+^. A paper strip pre-stained with BT solution was further fabricated and it also showed excellent sensing ability toward Cu^2+^ with a detection limit as low as 10^−6^ M with the naked eye, which represents better portability and operation simplicity that is favourable for on-site analysis of Cu^2+^ in water.

## 1. Introduction

Copper (Cu), as the third-most abundant transition metal after Fe and Zn in the human body, has important physiological roles in many biological systems [1–3]. However, overloading of Cu in organisms can cause serious danger, resulting in not only liver and kidney damage, but also severe oxidative stress and neurodegenerative diseases [4–10]. Considering this issue, the maximum permissible level of Cu^2+^ in drinking water has been determined to be 20 µM by US Environmental Protection Agency [11]. Therefore, it is essential to provide an appropriate method for recognition and determination of Cu^2+^ in water. Some colorimetric and fluorescent Cu^2+^ probes have already been reported with high sensitivity and selectivity [12–20]; however, most of them are operated in solution, which is not convenient for on-site analysis. On the other hand, solid-state sensors have become numerous in the last several decades, such as dipstick and lateral-flow assays, which are based on the blotting of analytes onto a paper pre-stained with probes [21,22]. The best-known example is the pH strip which is widely used to enable quick colorimetric response to different pH solutions. These formats have gained great popularity due to their feasible readout, good portability and operation simplicity. As a result, developing a Cu^2+^ strip, which can easily respond to different Cu^2+^ concentrations is really meaningful and valuable. It enables detection of Cu^2+^ by the naked eye with only a paper substrate.

**Figure 1.**
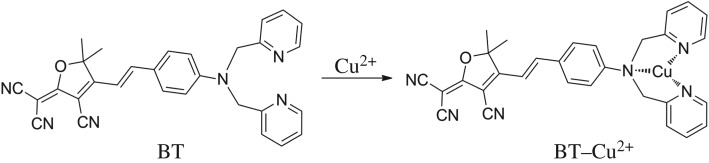
The recognition of BT toward Cu^2+^.

Bis(2-pyridylmethyl)amine (BPA) moiety had been used as a binding group to Cu^2+^ with good selectivity by Tian *et al.* [23] and Qin *et al*. [24] where their purposes were to detect pyrophosphate anion using the complex BPA–Cu^2+^. Because of its paramagnetic nature, Cu^2+^ usually leads to fluorescence quenching of the bonded fluorophore, resulting in fluorescent signal ‘turn-off’. Meanwhile, the electron-donating ability of the N atom in amine is seriously weakened when coordinated to Cu^2+^, leading to reduced intramolecular charge transfer (ICT) effect. As a result, the absorption spectrum of the molecule will be altered and enable colorimetric sensing of Cu^2+^. 2-(3-Cyano-4,5,5-trimethylfuran-2(5*H*)-ylidene)propanedinitrile (TCF), a well-known strong electron acceptor, can easily generate rather narrow band gap when connected to a strong electron donor with good conjugation, leading to long wavelength absorption and emission which is favourable for chemosensing due to reduced background interference. BT ((*E*)-2-(4-(4-(bis(pyridin-2-ylmethyl)amino)styryl)-3-cyano-5,5-dimethylfuran-2(5H)-ylidene)malononitrile) constructed by BPA and TCF moieties has been used as probe for Ni^2+^ in pure organic solvent CH_3_CN [25]. In this paper, we found new applications of BT that showed selective response to Cu^2+^ both in ethanol–water solution and on a paper strip (figure 1). When coordinated with Cu^2+^, the electron-donating ability of aniline in BPA unit is decreased, so the push–pull character of the dye is weakened, resulting in blue-shift of the absorption spectra, which enables the colorimetric sensing of Cu^2+^ with the naked eye. At the same time, Cu^2+^ leads to fluorescence quenching of the probe, which made BT a fluorescence ‘turn-off’ sensor for Cu^2+^. We further loaded BT to a common filter paper to fabricate a Cu^2+^ strip, and successfully realized the detection of Cu^2+^ in the form of a paper probe.

## 2. Material and methods

### 2.1. Materials and instruments

All reagents and solvents were commercially purchased, and the solvents were used after appropriate distillation or purification. The intermediates BPA, 4-(bis(pyridin-2-ylmethyl)amino)benzaldehyde (BPA-CHO) and TCF were synthesized according to the literature [26–28]. Stock solutions of compound BT (1 mM) were prepared in dimethylsulfoxide, then diluted to 10 µM in ethanol/HEPES (1 : 4 v/v) buffer (pH 7.2). All solvents used in the test were chromatographically pure. UV–visible absorption spectra were recorded on a Schimadzu 160A spectrophotometer. Fluorescence spectra were recorded on a Hitachi F-4500 spectrometer. The pH measurements were made with a Sartorius basic pH-meter PB-10. ^1^H NMR spectra were recorded on Bruker Ascend 400 MHz spectrometers, and ^13^C NMR spectra were recorded on 100 MHz spectrometers. Mass spectra were recorded on an Ion Spec 4.7T FTMS instrument.

### 2.2. Synthesis of BT

**Scheme 1.**
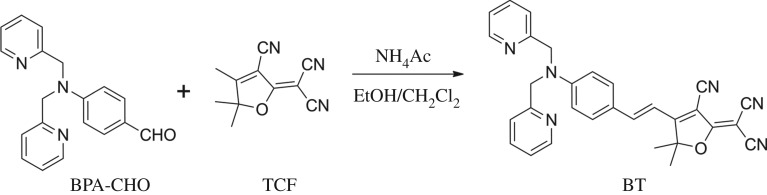
The synthetic route of BT.

The synthesis route of BT is shown in scheme 1. BPA-CHO (0.15 g, 0.50 mmol), TCF (0.11 g, 0.55 mmol) and ammonium acetate (0.046 g, 0.60 mmol) were stirred overnight in the dark under argon at room temperature in a mixture of ethanol (1 ml) and dichloromethane (1 ml). The solution rapidly turned from pale yellow to dark red. The mixture was diluted in water, extracted with dichloromethane and dried over anhydrous NaSO_4_. Then the solvent was removed under reduced pressure. The desired residue was purified by column chromatography on silica gel using EtOAc/petroleum ether (1/2, v/v) as the mobile phase to afford compound BT as amorphous black solid (0.21 g, 87%). ^1^H NMR (400 MHz, CDCl_3_) δ [ppm]: 8.62 (d, *J* = 4 Hz, 2H), 7.67 (t, *J* = 8 Hz, 2H), 7.58 (d, *J* = 16 Hz, 1H), 7.49 (d, *J* = 12 Hz, 2H), 7.27–7.20 (m, 4H), 6.82 (d, *J* = 12 Hz, 2H), 6.74 (d, *J* = 16 Hz, 1H), 4.94 (s, 4H), 1.73 (s, 6H). ^13^C NMR (100 MHz, CDCl_3_) δ [ppm]: 176.14, 174.28, 156.82, 152.75, 150.10, 148.04, 137.10, 132.11, 123.14, 122.69, 120.84, 113.27, 112.50, 111.70, 111.19, 109.74, 97.02, 95.38, 57.24, 55.27, 26.69. ESI-HRMS: [M+H]^+^, [C_30_H_25_N_6_O]^+^, calcd, *m/z* = 485.20; found, *m/z* = 485.23.

## 3. Results and discussion

### 3.1. Selectivity

**Figure 2.**
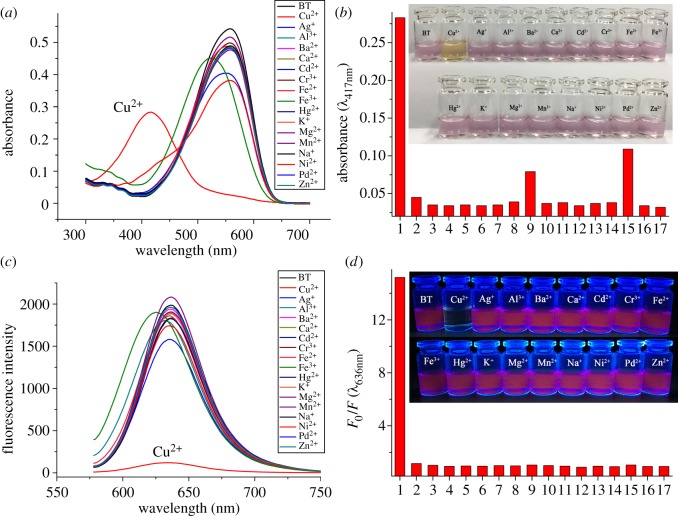
(*a*) Absorption spectra, (*b*) the absorbance at 417 nm, (*c*) fluorescence spectra (*λ*_ex_ = 558 nm, *λ*_em_ = 636 nm) of the mixed solution of BT (10 µM, in EtOH/HEPES buffer = 1/4, pH 7.2) with 5 equivalents of different metal ions, and (*d*) the ratio of fluorescence intensity (at 636 nm) before (*F*_0_) and after (*F*) addition of metal ions. Insets for (*b*) and (*d*): colorimetric and fluorescence photographs of BT with various metal ions (5 equivalents): (1) Cu^2+^, (2) Ag^+^, (3) Al^3+^, (4) Ba^2+^, (5) Ca^2+^, (6) Cd^2+^, (7) Cr^3+^, (8) Fe^2+^, (9) Fe^3+^, (10) Hg^2+^, (11) K^+^, (12) Mg^2+^, (13) Mn^2+^, (14) Na^+^, (15) Ni^2+^, (16) Pb^2+^ and (17) Zn^2+^.

Solution of BT in ethanol/HEPES buffer was carefully prepared with a concentration of 10 µM. Owing to the ICT effect from the electron donor (BPA) to the electron acceptor (TCF), BT covered a wide absorption range (figure 2*a*). The absorption maximum was at 558 nm which made BT solution show light purple red colour. The fluorescence spectrum revealed a maximum emission at 636 nm, which is red fluorescence owing to its low bandgap. And the fluorescence quantum yield of BT in ethanol/HEPES (1 : 4 v/v) buffer (pH 7.2) was calculated to be 0.13 using rhodamine B as a reference. To test the selectivity of BT to Cu^2+^ ions, 16 kinds of other metal ions including Ag^+^, Al^3+^, Ba^2+^, Ca^2+^, Cd^2+^, Cr^3+^, Fe^2+^, Fe^3+^, Hg^2+^, K^+^, Mg^2+^, Mn^2+^, Na^+^, Ni^2+^, Pb^2+^ and Zn^2+^ were chosen as contrast. Five equivalents of metal ions were added separately to BT solution, then the absorption and emission spectra were measured immediately and the results are shown in figure 2. Among the metal ions studied, only Cu^2+^ could change the spectra obviously with absorption maximum blue-shifted by 141 nm to 417 nm with sharp contrast. The colour change could be observed obviously by the naked eye (figure 2*b* inset). The phenomenon was ascribed to the decreased ICT effect between BPA unit and TCF unit, owing to the strongly weakened electron-donating ability of aniline N atom in BPA upon coordination to Cu^2+^. Although Fe^3+^ can make the absorption peak blue-shifted by 31 nm, the colour change of solution was not so obvious in that it cannot be clearly distinguished by the naked eye. BT solution with Ni^2+^ showed a weak shoulder band at 417 nm; however, the absorption maximum was still at 558 nm and the colour of solution was not changed. These results clearly demonstrated that BT was highly selective towards Cu^2+^ in colorimetric method. The fluorescence changes of BT solution were also determined after addition of metal ions. As shown in figure 2*c*, only Cu^2+^ could quench the fluorescence emission of BT efficiently with fluorescence intensity decreased by 15 times. Other metal ions did not affect the fluorescence obviously, except that Ag^+^ showed certain disturbance. There may be some interactions between pyridine unit and Ag^+^, thus photon-induced electron transfer may occur and affect the fluorescence emission of BT. The influence of Ag^+^ or other metal ions except Cu^2+^ on fluorescence of BT is not so obvious that it cannot be perceived by the naked eye when the solution was irradiated by a UV lamp with light of 365 nm as shown in figure 2*d*. Therefore, BT could also act as a fluorescence turn-off probe for Cu^2+^ with good selectivity.

### 3.2. Anti-interference

**Figure 3.**
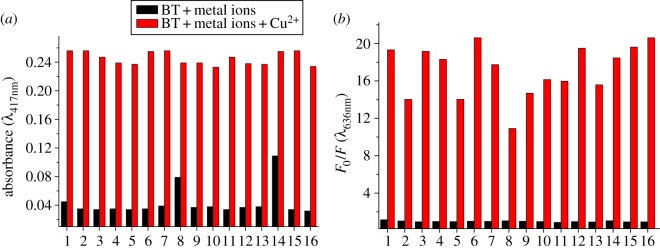
(*a*) Absorption spectra profile and (*b*) fluorescence spectra profile of BT (10 µM) in EtOH/HEPES buffer (1/4) solution with 5 equivalents of each metal ion, followed by 5 equivalents of Cu^2+^. M^*n*+^: (1) Ag^+^, (2) Al^3+^, (3) Ba^2+^, (4) Ca^2+^, (5) Cd^2+^, (6) Cr^3+^, (7) Fe^2+^, (8) Fe^3+^, (9) Hg^2+^, (10) K^+^, (11) Mg^2+^, (12) Mn^2+^, (13) Na^+^, (14) Ni^2+^, (15) Pb^2+^ and (16) Zn^2+^.

Competition experiment was performed to further confirm the selectivity of BT toward Cu^2+^. As shown in figure 3*a*, although Fe^3+^ can make the absorption peak slightly blue-shift and Ni^2+^ showed some background absorbance, they still did not affect the colorimetric recognition ability of the probe to Cu^2+^. As shown in figure 3*b*, Cu^2+^ can quench the fluorescence of BT solution efficiently even if the other metal ions are present. And it is interesting to note that the coexistence of most of the metal ions except Fe^3+^ strengthened the recognition ability toward Cu^2+^, as the change in fluorescence intensity was enhanced. In general, the BT probe demonstrated good anti-interference ability when detecting Cu^2+^ in both colorimetric and fluorescent modes.

### 3.3. Coordination mode

**Figure 4.**
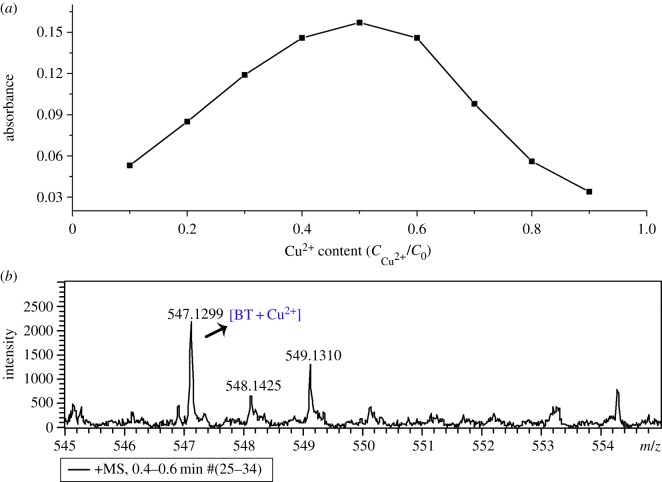
(*a*) Job's plot of BT to Cu^2+^ and (*b*) the mass spectrum of mixed solution of BT and Cu^2+^.

As the selectivity of the probe has been confirmed, to further understand the recognition nature of BT to Cu^2+^ ions, we investigated the coordination mode between them. The stoichiometry for the binding between BT and Cu^2+^ was studied by Job's plot. We maintained the total concentration (*C*_0_) of BT and Cu^2+^ unchanged, then altered the Cu^2+^ content ($C_{\text{C}{\text{u}}^{2+}}/C_{0}$) and recorded the absorption spectrum of each solution. The curve of absorbance at 417 nm versus Cu^2+^ content is depicted in figure 4*a*. It revealed that BT–Cu^2+^ complex reached the maximum absorbance when the Cu^2+^ content was 0.5, indicating that there was most amount of BT–Cu^2+^ complex in solution at this ratio. In other words, BT coordinated to Cu^2+^ with 1 : 1 stoichiometry in ethanol/HEPES buffer (pH 7.2) solution. This coordination mode was further confirmed by mass spectrum of the mixed solution of BT and Cu^2+^ as shown in figure 4*b*, where the molecular ion peak of [BT–Cu^2+^] could be found (calcd: 547.1297; found: 547.1299).

### 3.4. Sensitivity

**Figure 5.**
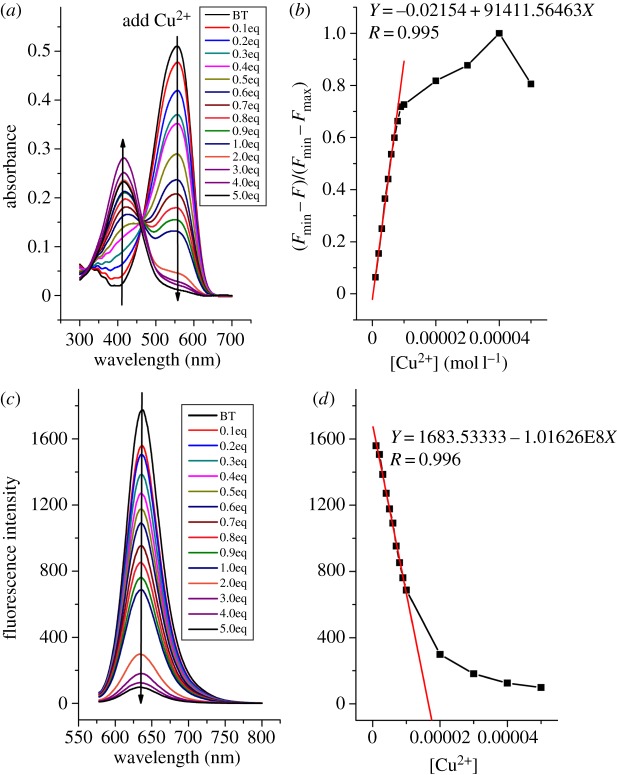
(*a*) UV–visible and (*c*) fluorescence spectra of BT solution (10 µM) with the addition of Cu^2+^. (*b*) Normalized absorption response (at 417 nm) and (*d*) fluorescence signal response to Cu^2+^ concentration.

The titration experiment was performed by adding various amounts of Cu^2+^ to BT solution, and both the absorption and fluorescence spectra were recorded. As shown in figure 5*a*, in the UV–visible spectra, the absorbance at 558 nm decreased, while that at 417 nm increased with the addition of Cu^2+^, revealing the formation of new complex between BT and Cu^2+^. From the normalized absorption signal response to the concentration of Cu^2+^ (figure 5*b*), the detection limit of colorimetric method was calculated to be 2.4 × 10^−7^ M [29]. In the fluorescence spectra, as shown in figure 5*c*, the fluorescence intensity decreased gradually with the addition of Cu^2+^, and the curve became smoother when more than 2 equivalents of Cu^2+^ was added. The fluorescence intensity was linearly related to the concentration of Cu^2+^ from 1 to 10 µM, and the fluorescence detection limit was 1.02 × 10^−7 ^M that was calculated on the basis of 3*σ*/*k* (*σ* is the standard deviation of the blank measurement and *k* is the slope of a plot of the fluorescence intensity versus Cu^2+^ concentration). The association constant of BT–Cu^2+^ was calculated to be 3.6 × 10^4^ M^−1^ using Benesi–Hildebrand analysis [30] (see electronic supplementary material).

### 3.5. pH sensitivity

**Figure 6.**
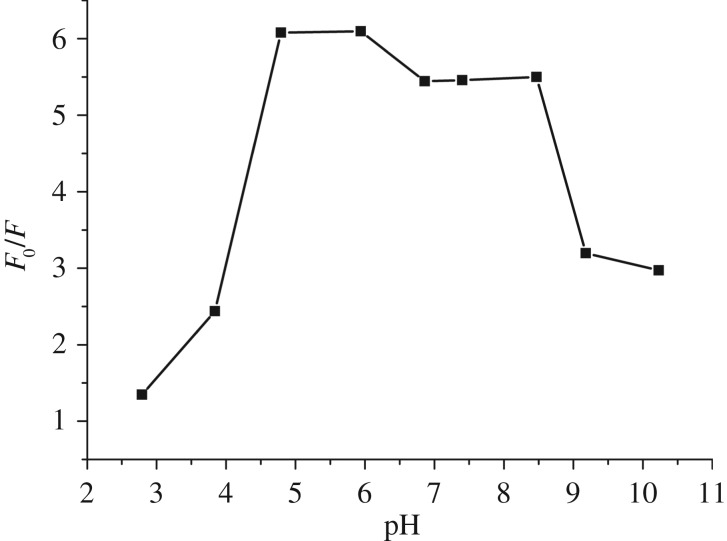
The performance of BT probe versus pH environment.

The performance of probe BT was tested in various pH environments. The fluorescence signal of BT solution before (*F*_0_) and after (*F*) addition of two equivalents of Cu^2+^ was separately collected. As shown in figure 6, the ratio of *F*_0_/*F* demonstrated obvious contrast with value more than 5 in the pH range of 5.0–8.5, manifesting that the probe can work well in this range. Too acidic an environment may protonate the aniline N and pyridine moiety which reduces the coordination ability of BPA group, and too basic an environment may reduce the free Cu^2+^ ions in solution and influence the sensitivity.

### 3.6. Cu^2+^ strip

**Figure 7.**
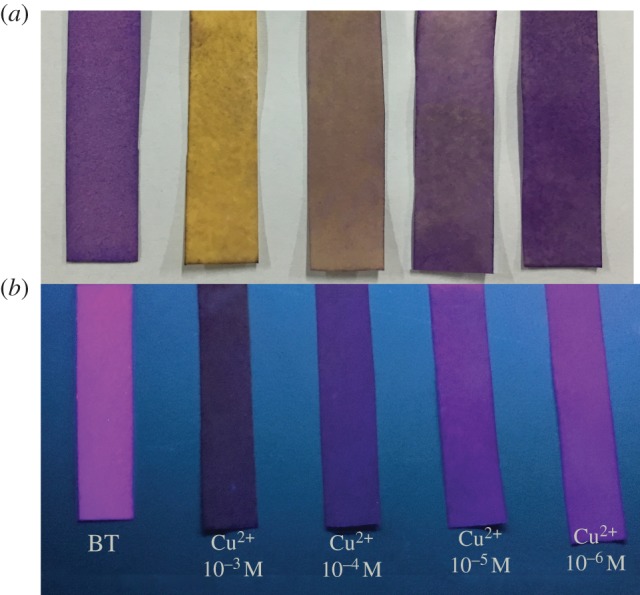
Naked eye detection of Cu^2+^ by the paper strip in (*a*) colorimetric and (*b*) fluorescent modes.

Probe BT showed high selectivity and sensitivity to Cu^2+^ in both colorimetric and fluorescent modes; however, it is not convenient enough to apply the probe in the form of solution for on-site analysis. If BT could be printed to a paper substrate to fabricate Cu^2+^ strip, the probe can demonstrate better portability and operation simplicity like the pH strip. Cu^2+^ strip was prepared by immersing a piece of tailed filter paper into BT solution (1 mM) in acetone for one minute, and then the paper was dried in air. For testing the performance of the strips, they were separately immersed into various concentrations of Cu^2+^ each for only one second. Obviously, the colour change could be observed by the naked eye immediately even if the concentration of Cu^2+^ was as low as 1 × 10^−5^ M (figure 7*a*). Fluorescence signal was also collected with the strip irradiated by a UV lamp, and the detection limit of this method can reach a level of 1 × 10^−6^ M (figure 7*b*). Different Cu^2+^ concentrations could give different signal changes in both colorimetric and fluorescence modes using this paper strip, revealing that the Cu^2+^ strip prepared here could work well.

## 4. Conclusion

A BT probe for the detection of Cu^2+^ constructed by BPA and TCF moieties was successfully synthesized with a wide absorption range and red emission. It showed high selectivity and sensitivity toward Cu^2+^ in ethanol/HEPES (1 : 4 v/v) buffer (pH 7.2) solution in both colorimetric and fluorescent modes. A paper strip was further fabricated easily by dipping common filter paper into BT solution, and the test results also showed good recognition ability to Cu^2+^, which makes the BT probe more portable and convenient. As a result, real-time and naked eye detection of Cu^2+^ ion could be realized by using a Cu^2+^ strip like the pH strip, which is useful in environment monitoring and water analysis.

## Supplementary Material

Electronic Supplementary Information

## Data accessibility

The synthesis of intermediates and some measurement information are presented in the electronic supplementary material of this article.
