# Supplementary material for: Colorimetric and fluorescent probes for real-time naked eye sensing of copper ion in solution and on paper substrate
Source: R Soc Open Sci. 2017 Nov 8;4(11):171161. doi: 10.1098/rsos.171161 (PMC5717676; doi:10.1098/rsos.171161)
Supplement: Electronic Supplementary Information [file rsos171161supp1.docx]

**Electronic Supplementary Information**

A colorimetric and fluorescent probe for real-time naked-eye sensing of copper ion in solution and on paper substrate

Dugang Chen,*^a,*^* Pengyu Chen,*^b^* Luyi Zong,*^b^* Yimin Sun,*^c^* Guangchao Liu,*^b^* Xianglin Yu,*^a^* and Jingui Qin*^b,*^*

*^a^* Key Laboratory of Green Chemical Process of Ministry of Education, School of Chemical Engineering & Pharmacy, Wuhan Institute of Technology, Wuhan 430205, China

*^b^* College of Chemistry and Molecular Science, Wuhan University, Wuhan 430072, China

*^c^* School of Materials Science and Engineering, Wuhan Institute of Technology, Wuhan 430205, China

*****Correspondence to:

D. Chen (E-mail: dg.chen@163.com) or J. Qin (E-mail: [jgqin@whu.edu.cn](mailto:jgqin@whu.edu.cn))

**1. Synthesis of intermediates**





Conditions: (a) NaOH(aq), H_2_O; (b) POCl_3_, DMF; (c) EtONa, EtOH.

*N,N-bis(pyridin-2-ylmethyl)aniline* (**BPA**)^1^: Aniline (1.67 g, 18 mmol), NaOH (3.24 g, 81 mmol) and Aliquat 336 (0.2 g) were dissolved in water (15 mL) and 2-picolyl chloride hydrochloride (5.91 g, 36 mmol) was added slowly at 0^o^C and stirred at room temperature for 24h. The mixture was diluted with 30 mL of water, extracted with dichloromethane, dried over anhydrous NaSO_4_. Then the solvent was removed under reduced pressure. The desired residue was purified by column chromatography on silica gel using CH_2_Cl_2_/petroleum ether(2/1, v/v) as eluent. BPA was obtained as brown solid (4.21 g, 85%).^1^H NMR (300 MHz, CDCl_3_) δ [ppm]: 4.81 (s, 4H), 6.69 (t, J=8.2 Hz, 3 H), 7.14 (t, *J*=6.3 Hz, 4 H), 7.25 (d, *J*=6.9 Hz, 2 H), 7.60 (t, *J*=7.65 Hz, 2H), 8.57 (d, J=4.5 Hz, 2H).

*4-(bis(pyridin-2-ylmethyl)amino)benzaldehyde* (**BPA-CHO**)^1^: POCl_3_ (1.83 mL, 19.6 mmol) and anhydrous DMF (25 mL) were stirred at 0 ^o^C under argon for 30 min. **BPA** (2.70 g, 9.8 mmol) was slowly added to the solution and it was maintained at this temperature for another 30 min. Then the mixture was warmed up to 90 ^o^C for 4h. The solution was poured into ice water and neutralized to pH 7 by adding NaOH solution (5%) dropwise. Then the mixture was extracted with CH_2_Cl_2_, washed with water for 3 times, and dried over anhydrous NaSO_4_. The crude product was purified by column chromatography on silica gel using Acetone/petroleum ether(1/3, v/v) as eluent to give BPA-CHO as pale solid (2.41 g, 81%). ^1^H NMR (300 MHz, CDCl_3_) δ [ppm]: 4.83 (s, 4H), 6.80 (d, *J*=9.0 Hz, 2H), 7.22–7.28 (m, 4H), 7.65–7.71 (m, 4H), 8.63 (d, *J*=3.9 Hz, 2 H), 9.74 (s, 1H).

*2-(3-cyano-4,5,5-trimethylfuran-2(5H)-ylidene)malononitrile* (**TCF**)^2^: To 1.80 g of sodium ethoxide (26 mmol, 0.30 equiv) dissolved in 12 mL of absolute EtOH in a room temperature water bath was added 3-hydroxy-3-methyl-2-butanone (9.0 g, 88 mmol) and 12 g freshly distilled malononitrile (181 mmol, 2.05 equiv) with stirring. After 1 h 30 mL of absolute EtOH was added and heated at reflux for 1 additional hour. This is cooled in a refrigerator and the solid filter, washed with a minimal amount of cold EtOH, and then air dried giving a first crop of 12.5 g of off-white crystalline solid (70% yield). Concentration of the filtrate and cooling gave a second crop of 0.8 g product (total yield 74%). ^1^H NMR (400 MHz, CDCl_3_) δ [ppm]: 2.36 (s, 3H), 1.63 (s, 6H);

**2. General procedure for analysis**

0.1 mmol of each inorganic salt was dissolved in distilled water (10 mL) to afford 10 mM aqueous solution. The stock solutions could be diluted to desired concentrations with water when needed, and the pH values were adjusted by the addition of concentrated solution of HCl with the aid of a pH meter. All measurements were made at room temperature. All spectra were obtained in a quartz cuvette (path length = 1 cm).

**3. The change of absorbance at 417 nm and 558 nm as the addition of Cu^2+^**


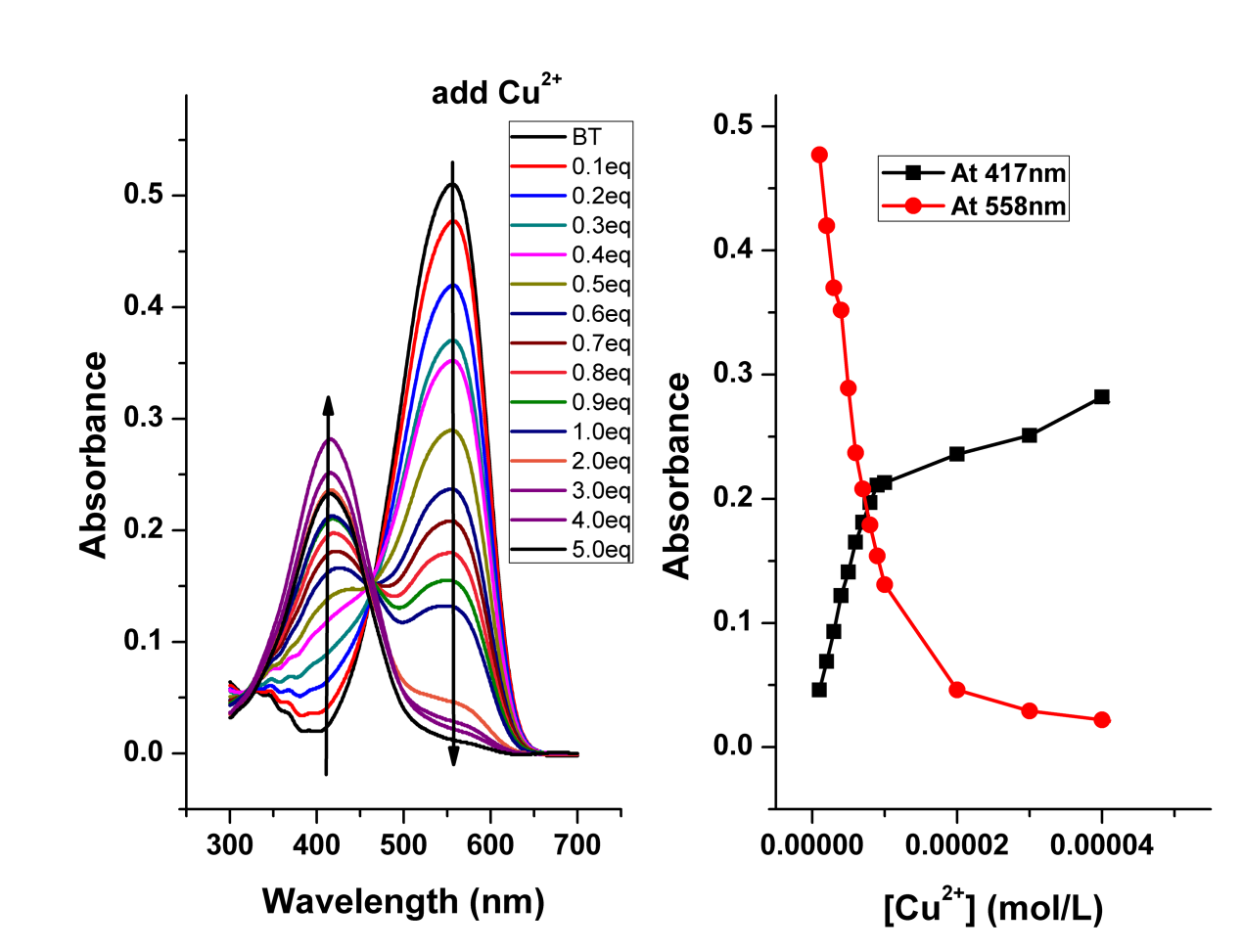


Fig. S1 The change of UV-vis spectra as the addition of Cu^2+^ to **BT** solution (10 μM).

**4. Calculation of σ**

σ = 10.37, for calculating detection limit.


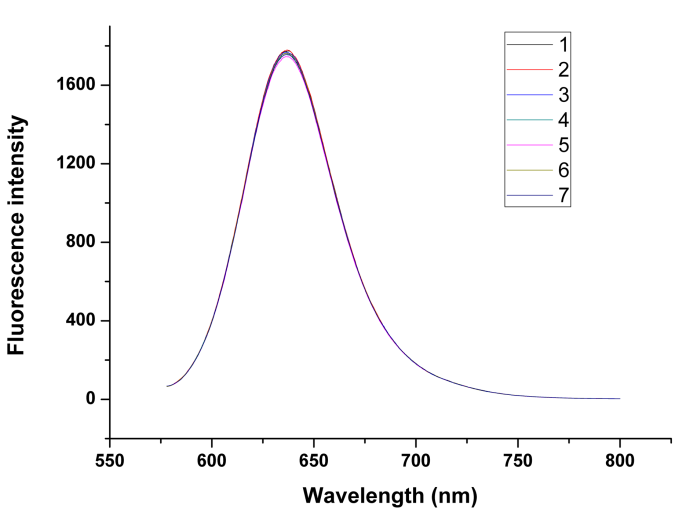


Fig. S2 Measurements of fluorescence spectra of blank solution for σ calculation.

**5. Calculation of association constants**

The association constant between probe **BT** and Cu^2+^ ions was analyzed using the fluorescence data and was calculated employing Benesi-Hildebrand.

$$\frac{1}{F_{0}-F}=\frac{1}{K\left( F_{0}-F_{\max} \right)\left[ \mathrm{Cu}^{2+} \right]}+\frac{1}{F_{0}-F_{\max}}$$

Where K is the association constant, F_0_ is the fluorescence intensity of probe **BT**, F is the fluorescence intensity of [**BT**-Cu^2+^] complex, and F_max_ is the maxima fluorescence intensity of the complex in the linear range.





Fig. S3 Benesi-Hildebrand curve

**Reference**

1. Y. Li, X. Dong, C. Zhong, Z. Liu and J. Qin, *Sens. Actuator, B*, 2013, **183**, 124-128.

2. M. Wu, K. Li, C. Li, J. Hou and X. Yu, *Chem. Commun.*, 2014, **50**, 183-185.
